# Letter from the Editor-in-Chief

**DOI:** 10.19102/icrm.2017.080203

**Published:** 2017-02-15

**Authors:** Moussa Mansour


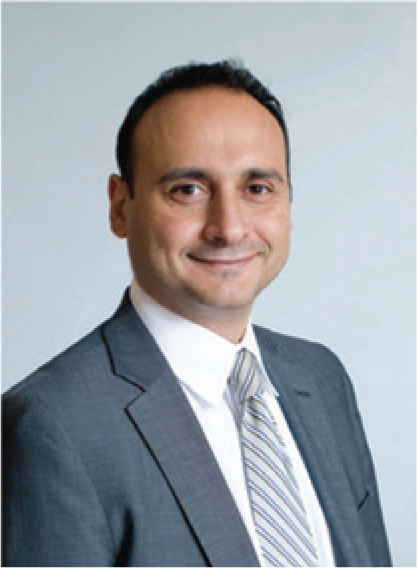


Dear Readers,

This issue of the *Journal* contains many interesting articles. I would like to highlight the one by Drs. Sze and Bahnson, titled “Pulmonary Vein Isolation Lesion Set Assessment During Radiofrequency Catheter Ablation for Atrial Fibrillation.” In this review, the authors discuss different techniques for confirming pulmonary vein isolation (PVI) during ablation for atrial fibrillation (AF). These include electrogram pattern recognition; the use of differential pacing to separate local from far-field signals; pace-capture at ablation sites; and the injection of adenosine and isoproterenol. In addition, they also discuss the role of lesion assessment technologies, such as MRI and acoustic radiation force impulse elastography.

The most important point in this article is the emphasis on durable PVI as the cornerstone of AF ablation. Over the past decade, the debate focus has shifted from whether or not PVI is important, to what technique is best to confirm a long-lasting isolation of the pulmonary veins. Another central point in this article is the underscoring of the importance of lesion assessment tools as critical components of a successful ablation procedure. Lesion assessment remains a major unmet need in the field of cardiac electrophysiology. Recently-developed indices combining input from ablation power, duration, contact force, impedance drop and catheter orientation are very promising and have the potential to significantly improve our ability to estimate adequate lesion formation; however, these indices are indirect surrogates for true lesion assessment and thus, are not necessarily optimally effective. More direct lesion assessment tools are also currently under consideration; these include dielectric sensors, ultrasound imaging, tissue temperature measurement and near-infrared spectroscopy.

In addition to lesion assessment, techniques to limit damage to collateral structures, such as the phrenic nerve and the esophagus, are also important to ensure durable PVI. Tools that help to avoid the occurrence of injury to structures adjacent to the left atrium not only improve the safety of the procedure, but also improve its efficacy by allowing for the delivery of RF energy with more power, higher contact force and at a longer duration. One promising technology in this area is esophageal retraction, which has the potential to eliminate the risk of esophageal injury and improve the success rate of PVI.

I hope that you enjoy reading this issue of the *Journal,* and that you find its content helpful in your practices.

Sincerely,


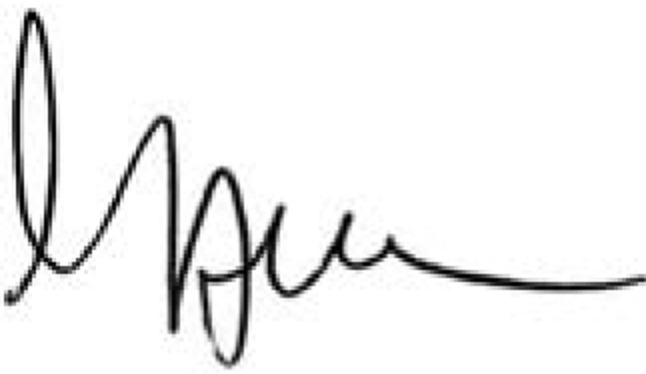


Moussa Mansour, MD, FHRS, FACC

Editor-in-Chief

The Journal of Innovations in Cardiac Rhythm Management

MMansour@InnovationsInCRM.com

Director, Cardiac Electrophysiology Laboratory

Director, Atrial Fibrillation Program

Massachusetts General Hospital

Boston, MA 02114

